# Making a Bad Situation Worse: An Invasive Species Altering the Balance of Interactions between Local Species

**DOI:** 10.1371/journal.pone.0152070

**Published:** 2016-03-24

**Authors:** Thayná Jeremias Mello, Alexandre Adalardo de Oliveira

**Affiliations:** 1 Departamento de Ecologia, Universidade de São Paulo, São Paulo-SP, Brazil; 2 Fernando de Noronha Marine National Park, Instituto Chico Mendes de Conservação da Biodiversidade, Fernando de Noronha-PE, Brazil; Estacion Experimental de Zonas Aridas - CSIC, SPAIN

## Abstract

Biological invasions pose a significant threat to biodiversity, especially on oceanic islands. One of the primary explanations for the success of plant invaders is direct suppression of competitors. However, indirect interactions can also be important, although they are often overlooked in studies on biological invasion. The shrub *Leucaena leucocephala* is a widespread island invader with putative allelopathic effects on the germination and growth of other species. We quantified the impact of *Leucaena* on plant communities richness on an oceanic Brazilian island and, through nursery experiments, investigated the potential for allelopathic effects on the germination of *Erythrina velutina*, a native species that is often absent from stands of *Leucaena*. Additionally, in a manipulative field experiment, we examined the direct and indirect effects (mediated by the native species *Capparis flexuosa*) of the invader on the development of *Erythrina*. The species richness in invaded sites was lower than in uninvaded sites, and *Capparis* was the only native species that was frequently present in invaded sites. In the nursery experiments, we found no evidence that *Leucaena* affects the germination of *Erythrina*. In the field experiments, the odds of *Erythrina* germination were lower in the presence of *Leucaena* litter, but higher in the presence of *Leucaena* trees. However, the survival and growth of *Erythrina* were considerably inhibited by the presence of *Leucaena* trees. The isolated effect of native *Capparis* on the germination and growth of *Erythrina* varied from positive to neutral. However, when *Capparis* and *Leucaena* were both present, their combined negative effects on *Erythrina* were worse than the effect of *Leucaena* alone, which may be attributed to indirect effects. This study provides the first empirical evidence that the balance of the interactions between native species can shift from neutral/positive to negative in the presence of an exotic species.

## Introduction

The key roles of interspecific plant interactions in shaping community composition and structure have long been recognised and demonstrated in experiments [1, 2]. Plant interactions can be resource or non-resource mediated, with either positive or negative effects on an organism’s fitness. Positive interactions occur when plants protect one another from herbivores or from extreme climatic conditions, or when they facilitate access to resources through hydraulic lift or nitrogen assimilation [3]. Negative interactions include resource competition and allelopathy, a mechanism in which chemical substances derived from decomposing litterfall or released by plant roots or leaves affect the germination and growth of neighbours [4]. Different types of plant-plant interactions can occur simultaneously, with variable outcomes for an organism’s fitness and for the sake of the local community [5].

Interactions often involve more than two interacting species, and webs of positive and negative effects in which one species can influence a second species either directly and/or indirectly through a third species can be produced [6]. These indirect effects are usually difficult to predict, detect and quantify but may play a fundamental role in structuring communities [7, 8]. Indirect effects take place through the concatenation of direct interactions and through the modification of interactions between pairs of species. Through a mediator species, indirect effects can offset, exacerbate or modify the direct effects of interactions [9].

Given the complexity of these webs of interactions, indirect interactions among native and invasive plants can also be an important component contributing to the net effects of invasive plants [10]. The addition of an exotic species to a community can disrupt its organisation and affect native species if the exotic species modifies the interactions between native plants and their enemies, mutualists and competitors [7]. These modified interactions may provide competitive advantages that influence the success of the introduced species [7, 11].

Invasion by exotic species represents a major threat to native communities, especially on oceanic islands [12]. There is currently no agreement regarding why islands are more prone to invasion [13], although the important role of biotic interactions with the native flora has been recognised in the invasion of islands [14]. Additionally, a review of invasions on islands concluded that only nine out of 383 alien species were consistently dominant on at least 50% of the islands where they were present [14]. One of these nine species is the leguminous shrub or tree species *Leucaena leucocephala* (Lam.) de Wit., which is native to Mexico. *L. leucocephala* is the third most widespread invader on oceanic islands [14] and belongs to one of the 40 most invasive genera of angiosperms in the world [15].

It has been suggested that the extremely high invasive potential of *L. leucocephala* may be attributed to the production of allelopathic substances that suppress the germination of seedlings of other species [16]. However, the majority of studies that have demonstrated allelopathic effects of *L. leucocephala* have involved laboratory bioassays and have used agricultural crops as the target species [16–19], which may have resulted in overestimation of these effects relative to what actually occurs in nature [20]. Research efforts to evaluate the allelopathic effects of invasive plants on native species with which they interact in nature may contribute to our understanding of the mechanisms of invasion [20].

In the 1940’s, *L. leucocephala* was introduced to Fernando de Noronha, an oceanic Brazilian island, and this species currently occupies extensive areas of the island. In general, introductions of plant species on islands result in an increase in species richness [12]. However, studies on plant regeneration in *L. leucocephala* plantations in Mexico have observed a reduction in species richness in areas occupied by *L. leucocephala*[21]. Preliminary field observations conducted on Fernando de Noronha have indicated that the exotic species appears to exclude most native species, and only a few native species persist in the patches where *L. leucocephala* is dominant. In contrast, several native species co-occur in the uninvaded communities on the island, in spite of the typical low species diversity of island communities compared with continental areas [22, 23]. This study aims to evaluate whether there is a negative impact of *L. leucocephala* on the species richness of Fernando de Noronha Island plant communities as well as to investigate the interactions of the invader with native species and to elucidate the mechanisms associated with the invasion of this species. In nursery experiments, we investigated whether there are allelopathic effects of the soil and the litter from *L. leucocephala* stands on the germination of the focal species, a common native species that is often absent from invaded areas. Through a manipulative field experiment, we examined the interactions of *L. leucocephala* with this native focal species both directly and indirectly via a mediator consisting of a native species that is persistent in invaded areas.

## Materials and Methods

### Ethics Statement

This research was conducted in the Brazilian Fernando de Noronha National Park and Environmental Protection Area under permission by ICMBio—Chico Mendes Institute for Biodiversity Conservation (SISBIO licence number 30161).

### Study site

Twenty-one islands of volcanic origin located in the South Atlantic Ocean make up the Fernando de Noronha archipelago (3°50′ S, 32°15′ W). The main island, Fernando de Noronha, has a 17 km^2^ total surface area. The distance between the archipelago and the nearest continent (South America) is approximately 360 km. The climate is tropical oceanic, with a mean annual temperature of 27^*o*^ *C*, and it experiences a strong influence of the trade winds. There are two distinct seasons in the area, a rainy season from February to July, followed by a pronounced water deficit from August to January, with a mean annual rainfall of approximately 1,400 mm and high interannual variability being observed [24].

Most of the island is covered by xeromorphic, seasonal, deciduous vegetation, with herbaceous, shrub and forest physiognomies. There is a historical record of 331 vascular plant species, 14 of which are endemic, but some are most likely extinct. Human settlement of the archipelago began in the 16th century, and activities such as deforestation, selective logging and forest fires have altered the native plant communities since that time. A large portion of the vegetation was removed approximately 200 years ago, when the island was used as a prison [25].

### Selected species

*Leucaena leucocephala* (hereafter, *Leucaena*) is a shrub or tree native to Mexico and Central America that grows to heights of 7–18 m. *Leucaena* is listed as one of the world’s 100 worst invasive alien species in the Global Invasive Species Database [26]. It was introduced to Fernando de Noronha as an alternative food supply source for livestock [24] because of its fast growth, drought tolerance and nitrogen assimilation capabilities. *Leucaena* form dense stands with a continuous canopy that can be monospecific or contain only a few other species [26].

The main native species that remains under *Leucaena* is *Capparis flexuosa* L. (Capparaceae). Alternatively, *Erythrina velutina* Willd. (Leguminosae) is a common native species on the island but rarely occurs together with *Leucaena* (see results for details). Thus, these three species were selected for our experiments.

*Erythrina velutina* (hereafter, *Erythrina*) is a pioneer tree that may grow up to 25 m tall. It is one of the most frequent [23] and widely distributed tree species on the island [27], in spite of being heavily logged during the first years of human settlement on the island [28]. Because of its wide distribution across the island and its potential use for the restoration of disturbed areas [29], this species was the focal species in all the experiments.

*Capparis flexuosa* (hereafter, *Capparis*) is a shrub that grows to a height of 2–4 m. It is one of the few evergreen species on the island. *Capparis* can be found in most vegetation types, especially in coastal areas [24], in open physiognomies as isolated shrubs or in the understory of forests. *Capparis* occurs in both invaded and uninvaded sites (see results) and was chosen as the mediator species for the field experiments.

### Invasion and community survey

We conducted an invasion survey in 176 plots with a radius of 20 m distributed systematically every 300 m across the entire island. At each plot, we attributed an ordinal ranking of the levels of *Leucaena* invasion. The ranking was as follows: 0, *Leucaena* not present (uninvaded site); 1, isolated *Leucaena* plant (only one plant within the plot); 2, scattered *Leucaena* plants (isolated plants dispersed within the plot); 3, *Leucaena* plants in groups (clusters of plants dispersed within the plot); 4, *Leucaena* plants forming mixed stands with other species (highly invaded site); and 5, pure *Leucaena* stands.

We assessed native species richness at both highly invaded and uninvaded sites, based on the map of the spatial distribution of the ranks of *Leucaena* described above. We randomly selected 10 sites among the highly invaded (rank 4) and 10 sites among the uninvaded (rank 0). We recorded the number and identity of native species with diameter at breast height (DBH) greater than 1 cm in circular plots with a radius of 3 m located in the center of each site.

### Nursery experiments

To examine the effects of soil from *Leucaena* stands on the germination of *Erythrina* seeds, we chose 10 sites and established plots both within a *Leucaena* stand and in a nearby uninvaded area. The uninvaded plot was always within 100 m of the *Leucaena* plot and did not differ in soil type. We collected 10 L of soil from each plot and removed the litter from the samples. Then we mixed and homogenized the soil to prepare each treatment. Then, we filled 10 trays with invaded and 10 with uninvaded soil type (a total of 20 trays) and sowed 35 seeds of *Erythrina* per tray. The seeds used in all of the experiments were collected from several trees across the island at the end of the fruiting period. We arranged the trays randomly in a nursery under homogeneous environmental conditions and watered the trays once per day. We recorded seed germination daily for 10 days, until no seedlings emerged for 3 consecutive days.

To examine the effects of *Leucaena* litter on the germination of *Erythrina*, we filled 20 trays with vermiculite, sowed 35 *Erythrina* seeds in each tray and arranged the trays randomly in an area with homogeneous environmental conditions in the nursery. We covered 10 trays with a 1 cm layer of *Leucaena* litter and 10 trays with a 1 cm layer of shredded filter paper. Shredded filter paper was used to mimic litter cover, including the retention of moisture by the litter, but without releasing chemicals. We watered the trays once per day and recorded seed germination daily for 10 days, until no seedlings emerged for 3 consecutive days.

### Field experiment

We designed a field experiment including four treatments: 1) a control, with *Leucaena* and *Capparis* removed (i.e., bare soil); 2) *Capparis* present and *Leucaena* removed; 3) *Leucaena* present and *Capparis* removed; and 4) both *Leucaena* and *Capparis* present (i.e., no removal). In the treatments involving removal, all trees and seedlings of the target species were removed from a 9 m^2^ area, with uprooting to prevent resprouting. Removal of new *Leucaena* seedlings was performed monthly for nine months.

**Fig 1:**
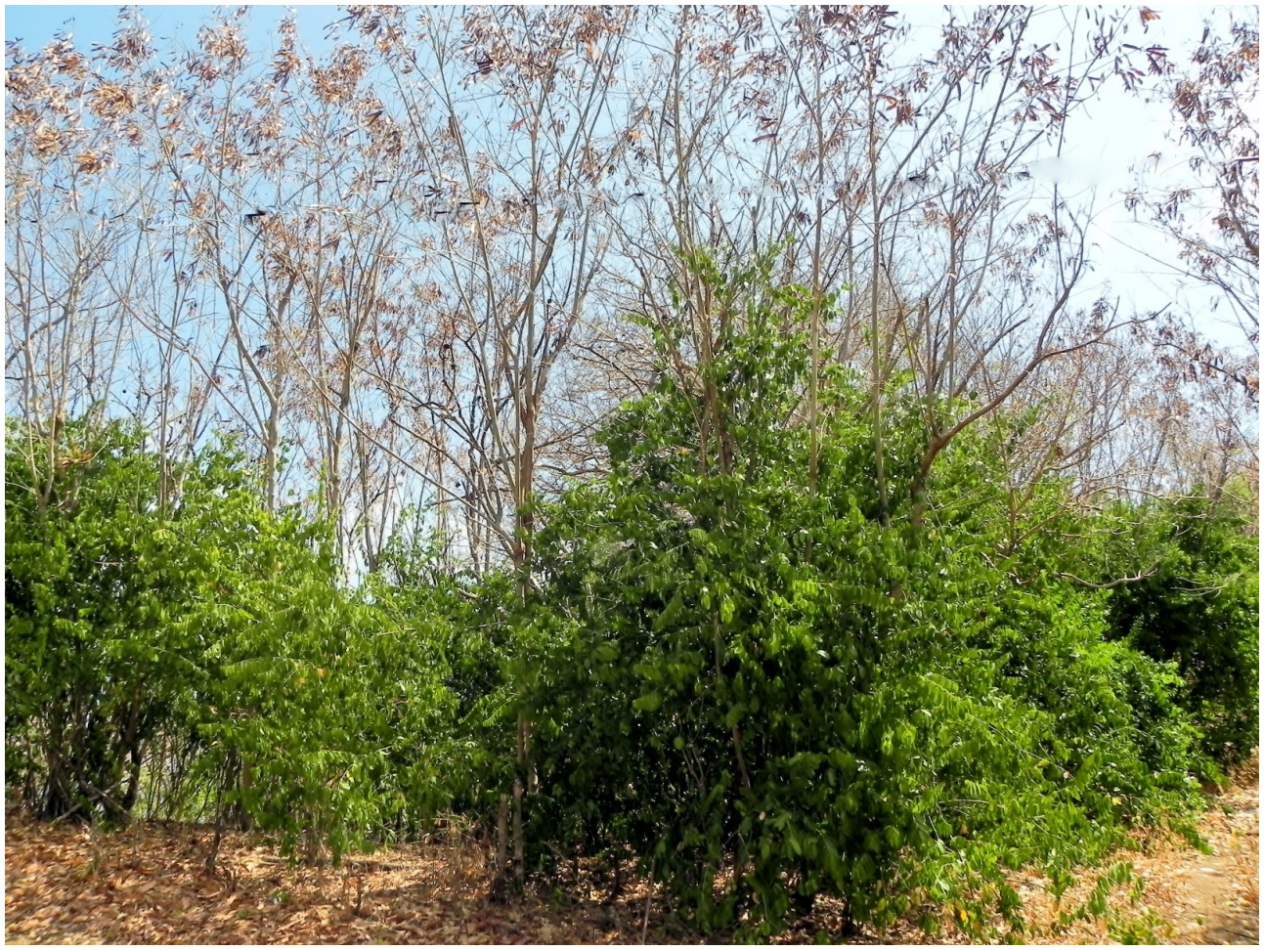
Coastal plain vegetation at the north shore of Fernando de Noronha island, Brazil. This vegetation, utilized in our experiments, is composed of associations of invasive *Leucaena leucocephala* (upper stratum) and native *Capparis flexuosa* (lower stratum). *C. flexuosa* is the most common free-standing woody plant in the Island, occurring in most areas invaded by *L. leucocephala* and in 90% of the uninvaded areas.

We installed 10 experimental blocks each one containing the four treatments assigned in a random fashion, with a minimum distance of 30 m between blocks, in an area on the north coast of the island where the current vegetation is mainly composed of associations of *Leucaena* with *Capparis* (Fig 1). This coastline extends for 1,700 m, with a width of 40 to 100 m, and is characterised by sandy soil and no significant variation in elevation.

In the beginning of the rainy season (April 2012), we sowed 50 seeds of *Erythrina* in each treatment, for a total of 200 seeds per block. We covered half of the seeds in each field treatment with a 1 cm layer of soil from the site itself and 1 cm layer of *Leucaena* litter, and we covered the other half of the seeds only with soil. We identified each seed with a flag to determine the fates of individual seeds. We first recorded germination at 7 days after planting, by which time most of the seeds that would germinate had germinated, and again at 30 days, after which no additional seedlings emerged.

We identified each seedling that germinated with a tag and recorded seedling survival seven times during the 9 months of the study in each field treatment. The interval between censuses varied from 30 to 60 days. We measured seedling heights at 9 months after the beginning of the experiment.

### Data analyses

To test for differences in species richness between invaded and uninvaded areas, we used a Monte Carlo Simulation approach [30]. We first calculated the observed mean difference between highly invaded and uninvaded plots and them performed 10,000 permutations, resampling the occurrence of species in those plots and keeping the number of ocurrences for each species fixed. For each permutation we recalculated the pseudo-value of mean difference between highly invaded and uninvaded plots. We then used the distribution of pseudo-values to calculate the probability that the observed difference in mean richness was expected in the null model of random occurences of species.

For the nursery experiments, we compared models that included the presence of *Leucaena* as a fixed effect and the trays as a random effect with null models that included only the random effect of the trays to evaluate the effects of soil and *Leucaena* litter on *Erythrina* seed germination.

For the germination field experiment, we evaluated the influence of *Leucaena*, *Capparis*, litter and their second- and third-order interactions as fixed effects in a generalized linear mixed effect model (GLMM). We used binomial error to model the proportion of germinated seeds and included the blocks as a random effect. To measure the partial effect of each variable on the odds of germination, we calculated the odds ratio and 95% confidence interval for each coefficient in the selected model. Odds ratios >1 indicate positive effects on germination, while odds ratios <1 indicate negative effects.

In the survival analysis of *Erythrina* seedlings we considered *Leucaena*, *Capparis* and their interactions as fixed effects. We model time to death with Weibull distribution and used seedlings alive at the end of the experiment as a right censored data and blocks as frailt model with Gaussian distribution for time variable [31]. For both germination and survival we used a model selection approach to evaluate all possible combination of factors and interactions and compared models in terms of the level of support from the observed data, considering both fit and complexity [32]. For all models we computed coefficients using the Maximum Likelihood Estimation (MLE) and used Akaike’s Information Criterion (AIC) for statistical inferences and considered models showing a *ΔAIC* < 2 equally plausible [33].

After 9 months, several plots and blocks exhibited 100% of seedling mortality. Therefore, we grouped all remaining seedlings in each treatment to evaluate their growth at the end of the experiment. We measured the height and the base diameter of each seedling. The pattern observed for the diameter and the height was the same, so we present only the results for the latter. During all the experiment we did not find any indication that height was biased by other factors besides our variables of interest and we used a bootstrap approach to generate conservative confidence intervals (99%) for the mean heights of the seedlings in each treatment and considered differences when the confidence intervals did not overlap.

All statistical analyses were conducted and graphics were generated in R 3.1.0 [34]. The GLMMs were formulated using the glmer function from the lmer package [35], survival analysis using survival package [31] and model selection was performed using the AICtab function in the bblme R package [36].

## Results

### Invasion and community survey

**Fig 2:**
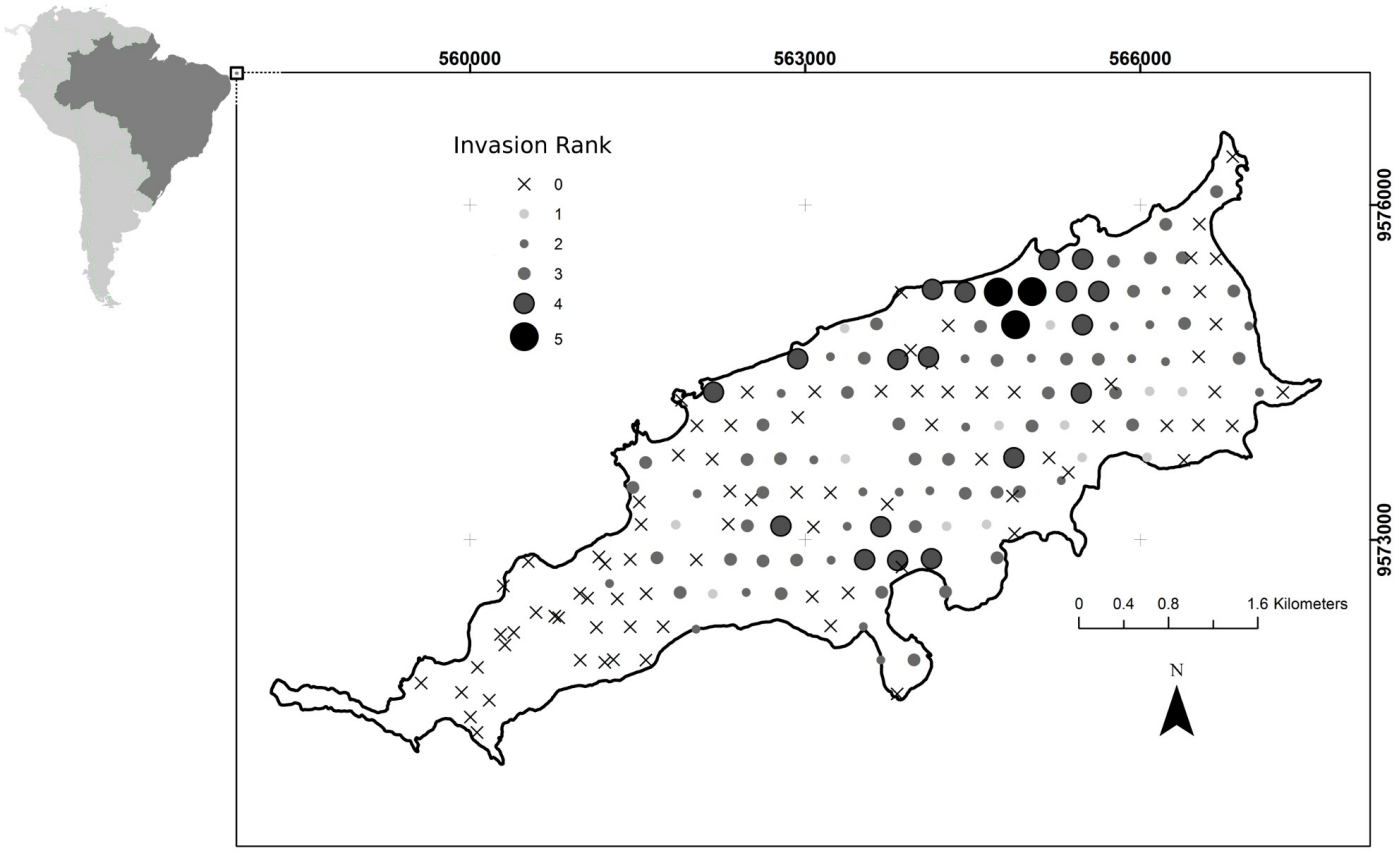
Invasion ranking of *Leucaena leucocephala* observed in 176 plots sampled in Fernando de Noronha Island, Brazil. 0, not present (uninvaded site); 1, isolated plant (only one plant within the plot); 2, scattered plants (isolated plants dispersed within the plot); 3, plants in groups (clusters of plants dispersed within the plot); 4, plants forming mixed stands with other species (highly invaded site); and 5, pure *L. leucocephala* stands.

**Fig 3:**
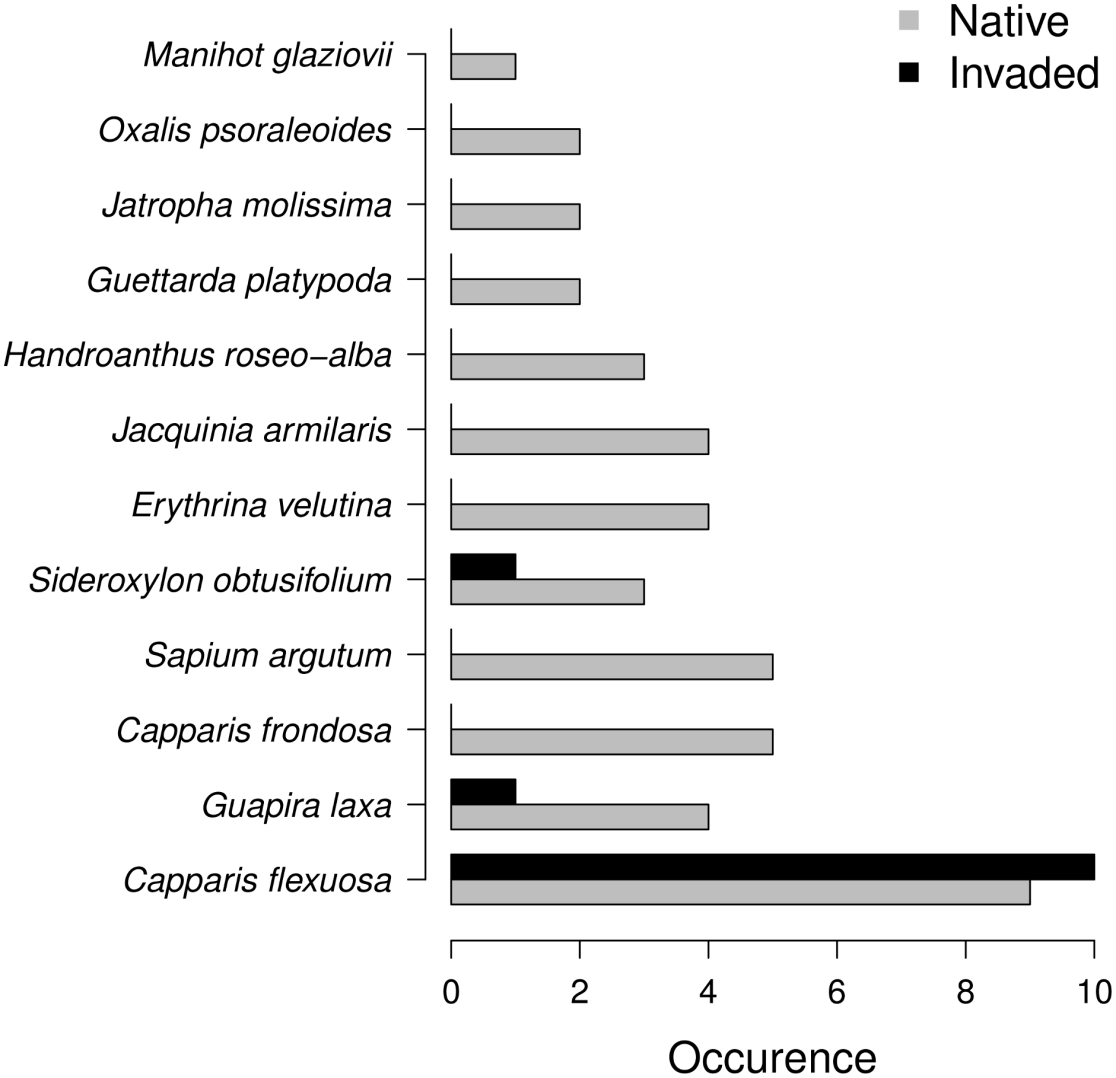
Frequency of native species in invaded areas and native forest sites at Fernando de Noronha Island, Brazil. Sites were randomly selected among the highly invaded and among the uninvaded (10 sites each). Number of free-standing woody species with diameter at breast height (DBH) greater than 1 cm was recorded in circular plots with a radius of 3 m located in the center of each site.

*Leucaena* was absent from only 39.5% of the plots sampled in the island, and 10.5% of the plots sampled were highly invaded, with *Leucaena* plants forming mixed stands with other species. In 1.7% of the plots *Leucaena* formed pure stands (Fig 2). The mean richness difference of tree species between invaded and uninvaded sites was significant (*p* < 0.001). In the areas highly invaded by *Leucaena*, but where native species were present, we recorded a total of three native species (mean of 1.2 ± 0.42 native species/site) and at uninvaded sites 12 native species (mean of 4.4 ± 1.78 native species/site). *Capparis* was the most common native species and was present in all of the invaded sites and in 90% of the uninvaded sites (Fig 3). The only species recorded in invaded sites other than *Capparis* were *Guapira laxa* (Netto) Furlan (Nyctaginaceae) and *Sideroxylon obtusifolium* (Humb. ex Roem. & Schult.) T.D. Penn. (Sapotaceae). *G. laxa* and *S. obtusifolium* were present in just 10% of the invaded sites, whereas 12 native species co-occurred with *Capparis* in the uninvaded sites.

### Nursery experiments

The number of seeds that germinated varied greatly between experiments but not between treatments. Trays with soil from invaded areas had a mean of 29.4 ± 3.7 (*mean* ± *sd*) germinated seeds per tray and uninvaded 26.7 ± 4.2. The treatment with *Leucaena* litter had a mean of 5.4 ± 1.8 and paper cover 4.5 ± 1.9 germinated seed per tray. In the soil experiment, the difference in the AIC between the model including the effect of *Leucaena* (coefficient estimate: 0.52 ± 0.30) and the null model was less than two (*ΔAIC* = 0.9); thus, the models were considered equally plausible. A similar result was observed for the litter experiment, where the difference in the AIC between the model including the effect of litter (coefficient estimate: 0.21 ± 0.22) and the null model was less than two (*ΔAIC* = 1.0). So, in both cases there is an indication of the positive effect of *Leucaena*’s litter and soil, but there is uncertainty about those effects.

### Field experiments

#### Germination

**Fig 4:**
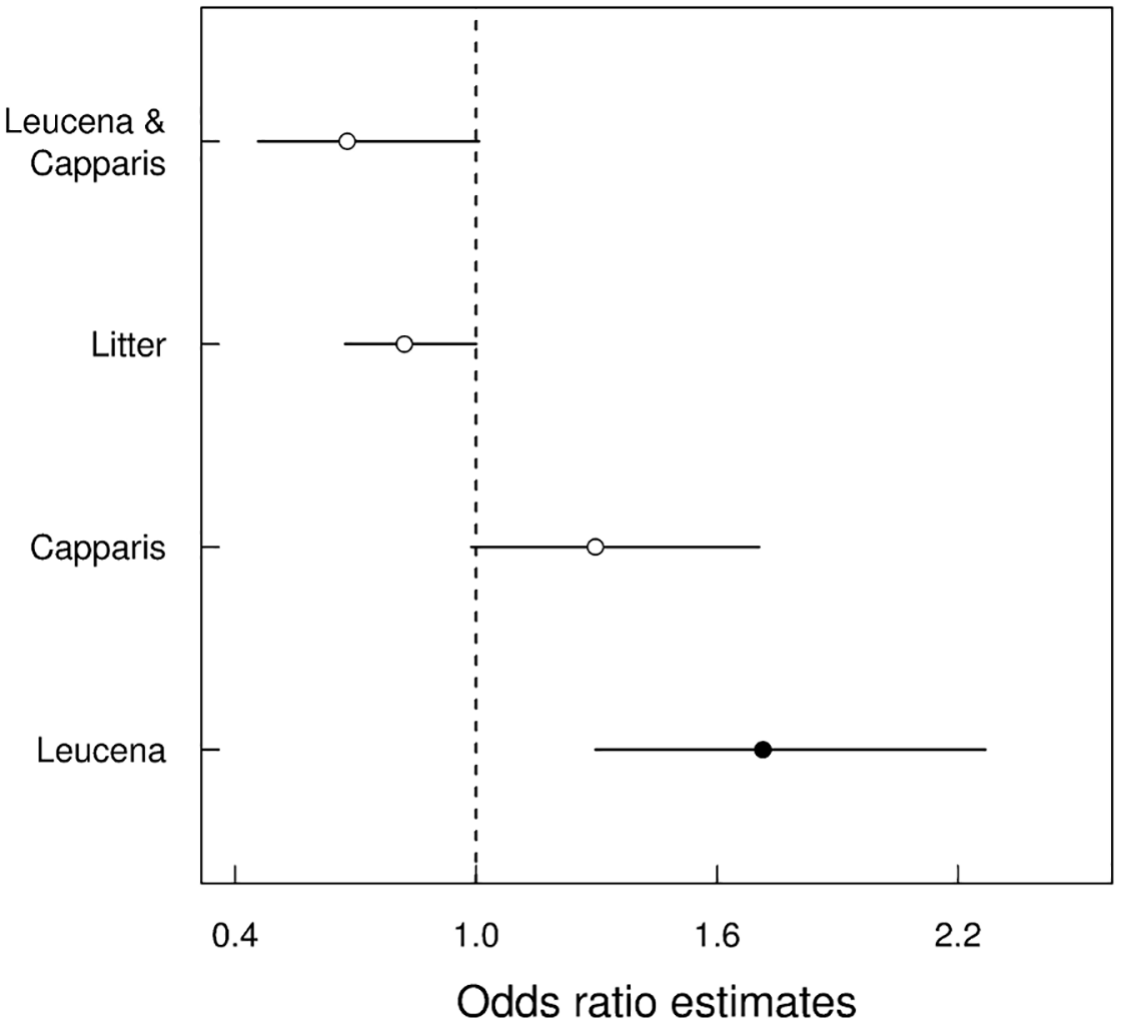
Odds ratios of model 5 (lowest AIC—Table 1), describing the germination of the native species *Erythrina velutina* in a field experiment. Circles show the odds ratios for each treatment, with the 95% confidence limit (CL) represented by the horizontal lines. Odds ratios that include a ratio of 1 within the 95% CL are indicated with empty circles.

Comparisons between the effects of each treatment and the control on the number of seeds that germinated indicated a high degree of variation among the blocks. Five out of the 16 models compared were equally plausible. The presence of *Leucaena* and *Leucaena* litter were the only effects found in all of the plausible models (Table 1). The selected models predicted that the presence of *Leucaena* increases the odds of germination by approximately 1.8-fold, while *Leucaena* litter has the opposite effect and decreases the odds of germination by approximately 0.8-fold. The importance of the effects of *Capparis* and of the second-order interactions was uncertain, but the model with the lowest AIC predicted a positive effect of *Capparis* and a negative effect of the *Leucaena*:*Capparis* interaction (Fig 4).

**Table 1:** Selected models for the effects of the treatments on the germination of *Erythrina velutina* in the field experiment, with the coefficient estimates (± standard error) of the parameters included in each model. The models were binomial generalised linear mixed models (GLMMs) with germination as the response variable, the presence of *Leucaena leucocephala* (leu), *Capparis flexuosa* (cap) or litter (lit) and their interactions as fixed predictor variables and the blocks as a random variable. AIC = Akaike’s Information Criterion; *ΔAIC* = AIC for each model—AIC for the best model.

Model parameters included						Δ*AIC*
leu	cap	lit	leu:cap	cap:lit	leu:lit	
0.54 ± 0.25	0.26 ± 0.14	−0.20 ± 0.10				0.0
0.34 ± 0.10		−0.20 ± 0.10				0.3
0.54 ± 0.14	0.26 ± 0.17	−0.10 ± 0.14	−0.39 ± 0.20	−0.19 ± 0.20		1.1
0.35 ± 0.10	0.07 ± 0.10	−0.20 ± 0.10				1.7
0.54 ± 0.25	0.26 ± 0.14	−0.20 ± 0.10			−0.07 ± 0.20	1.9

#### Survival

**Fig 5:**
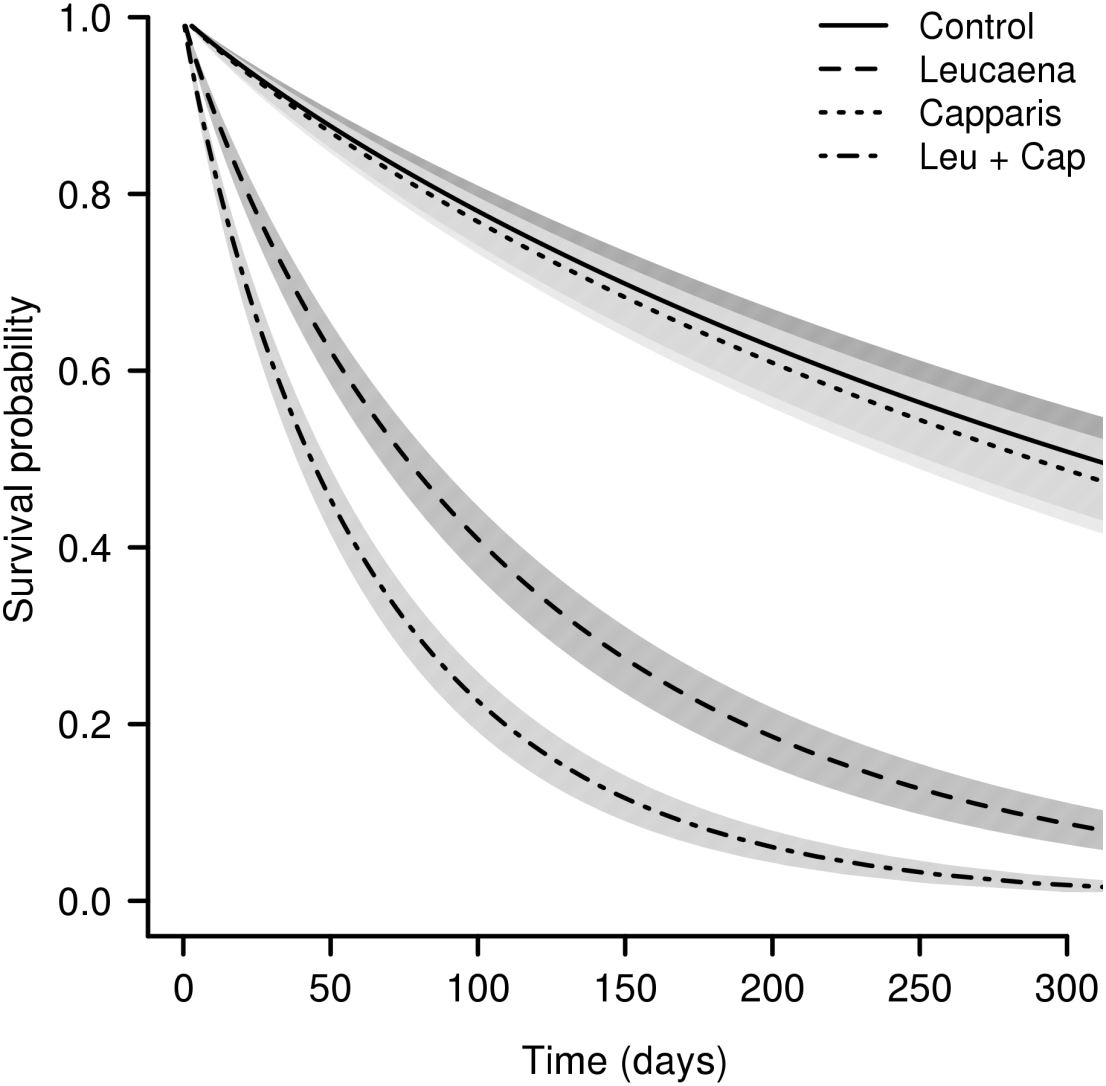
Survival function of *Erythrina velutina* seedlings predicted by survival model selected. Survival model with Weibull distribution and experimental blocks as frailt model with Gaussian distribution. Factor included in the most plausible model were presence of *Leucaena leucocephala* (leu), presence of *Capparis flexuosa* (cap), interaction (leu:cap). Shaded areas indicate the 95% predicted confidence interval.

After the first census (approximately 30 days after germination), the survival of *Erythrina* seedlings was higher in both the control (79%) and under *Capparis* (87%) than under *Leucaena* (58%) or *Leucaena* plus *Capparis* (39%). From the second census onwards (approximately 60 days after germination), the survival was lower for the *Erythrina* seedlings under *Leucaena* compared with the control. In contrast, there was no significant difference between the survival of the seedlings under *Capparis* versus the control. The lowest survival was observed for the seedlings under *Leucaena* plus *Capparis*. At the end of the experiment, the total survival rate was approximately 60% in the control and under *Capparis*, approximately 10% under *Leucaena* and approximately 3% under *Leucaena* plus *Capparis*. The selected survival model for *Erythrina* seedlings included the main effects of *Leucaena*, *Capparis* and interactions between these factors. The model without the interaction had *ΔAIC* > 10 (S1 Table). According to the selected model (Table 2), there was no effect of *Capparis*, a negative effect of *Leucaena* and a strong negative effect of the *Leucaena:Capparis* interaction on *Erythrina* seedling survival. The median survival time prediction in days and its confidence interval was 308(±56) for control, 288(±47) for *Capparis* presence, 76(±9) for *Leucaena* presence and 43(±5) for *Leucaena* and *Capparis* presence. In terms of median survival time the presence of *Leucaena* accelerate time to death by 4-folds, and *Leucaena* and *Capparis* together decrease the median time to death more than 7 times (Fig 5).

**Table 2:** Coefficient estimates and standard errors of the parameters included in the most plausible model for the effect of the treatments on survival time of *Erythrina velutina* seedlings in the field experiment. The model has survival time as Weibull distribution with experimental blocks as frailt model with Gaussian distribution. Model coefficients: control (int); presence of *Leucaena leucocephala* (leu), presence of *Capparis flexuosa* (cap), interaction (leu:cap) and log scale (lsc), a Weibull fixed parameter.

	Parameters				
	int	leu	cap	leu:cap	lsc
**Estimate**	6.131	-1.424	-0.078	-0.497	0.011
**Std. Error**	0.162	0.104	0.116	0.139	0.026

#### Growth

**Fig 6:**
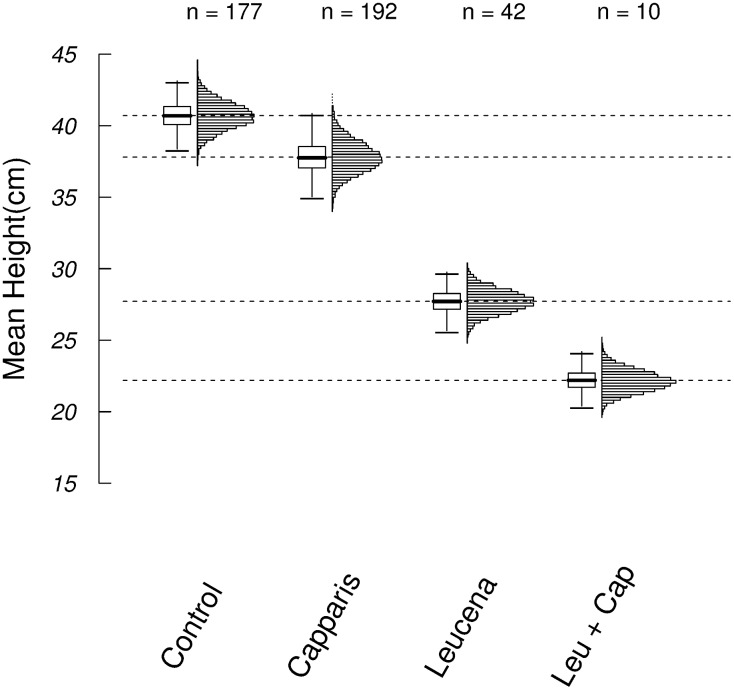
Heights of *Erythrina velutina* seedlings at 9 months after sowing in four different treatments. Horizontal dashed lines indicate the observed mean heights. The bars in the boxplots indicate 99% of the bootstrap interval; the boxes confine 50% of the bootstrap interval. The number of surviving seedlings at the end of the experiment for each treatment is indicated above the graph. Histograms show the distributions of the bootstrap values. Control: areas without *Leucaena leucocephala* and *Capparis flexuosa*, Cap+Leu: areas with *Capparis flexuosa* + *Leucaena leucocephala*.

Nine months after sowing, the height of the *Erythrina* seedlings that survived was greater in the control and under *Capparis* than under *Leucaena* and *Leucaena* plus *Capparis*. The distributions of the mean height values resulting from the permutation indicated differences between the heights of seedlings in the control treatment, under *Leucaena* and under *Leucaena* plus *Capparis* (Fig 6). The mean height of the *Erythrina* seedlings grown under *Leucaena* plus *Capparis* (22.2 cm) was 55% of the mean seedling height in the control areas (40.7 cm), whereas under *Leucaena*, the mean height (27.7 cm) was 68% of the control mean height.

## Discussion

The invasive species *Leucaena* is clearly associated with lower native species richness in Fernando de Noronha Island, an important National Park in Brazil. This exotic species is widely distributed on the island, densely covering most places where it occurs. It represents a threat to native tree species, and the area of the island covered by *Leucaena* increased in the last 20 years (T. Mello et al., unpublished data). The tree species richness was clearly lower (by 50%) in areas occupied by *Leucaena* compared with uninvaded sites. Moreover, we observed a tendency towards homogenization in the invaded areas, as the only native woody species present in most of the invaded areas was *Capparis*. *Capparis* is also a common species in uninvaded communities, where it co-occurs with many other native species.

The results of the nursery experiment did not demonstrate allelopathic effects of *Leucaena* on *Erythrina* germination, either for the soil or for the litter from the sites with *Leucaena*. This finding is contrary to the results of studies that have detected allelopathic effects of *Leucaena* on the germination of crop species (e.g., [18]). In fact, we found some evidences of positive effect, however, there was uncertainty associated with this effect due to the high variability in the response of germination. Therefore, this positive effect deserves further investigation. Additionally, although our results clearly demonstrate no allelopathic effect, it is possible that *Leucaena* releases harmful allelochemicals that do not remain in the soil for long after the invader is removed, which is the opposite of what happens under the “legacy effect” phenomenon [37].

In the germination phase of the field experiment, the presence of litter slightly decreased the odds of germination for the seeds compared with the control. Our results suggest that litter, as well as a inert substrate layer (e.g. shredded filter paper) act as a mechanical barrier that hindered the emergence of the seedlings [38], but we did not test this mechanism directly. The presence of *Leucaena* trees had a positive effect on germination, contrary to what we expected. This positive effect may have been related to an improvement in the local microclimate associated with the presence of *Leucaena* trees. The trees provide shade and act as a barrier against the wind, helping to retain moisture, which may, in turn, facilitate germination. Studies conducted with other species of the genus *Erythrina* have demonstrated that water is the most important factor for seed germination, whereas no differences in germination have been observed between seeds in the dark versus those exposed to light [39, 40]. Thus, by preventing desiccation, *Leucaena* may facilitate the germination of *Erythrina*. This positive effect in the field corroborates the nursery experiments and increases the evidence of no allelophatic effect of *Leucaena* on *Erythrina* germination.

The effect of *Leucaena* on *Erythrina* varies according to the life stage of the native plant. Although a positive effect of *Leucaena* trees on the germination of *Erythrina* seeds was found, their effect on the survival and growth of the seedlings was clearly negative. Despite facilitating germination, *Leucaena* prevents the development of the seedlings and reduces their survival. These findings suggest that, if there are phytotoxic effects of *Leucaena*, they act mostly during the establishment phase, rather than during the germination of seedlings under the *Leucaena* canopy. This pattern is similar to the one observed by Peguero and collaborators [41], who studied the establishment of seedlings under *Acacia pennatula* Benth, another leguminous species with putative phytotoxic effects, in a dry forest in Nicaragua.

The effect of *Capparis* on the survival and growth of *Erythrina* seedlings varied from positive to neutral depending on the age of the seedlings. The *Capparis* crown is denser and intercepts more light than the *Leucaena* crown. In addition, *Capparis* is an evergreen species, most likely requiring plentiful water, while *Leucaena* can be deciduous in the dry season. Thus, if the negative effect of *Leucaena* on the survival and growth of *Erythrina* seedlings was related mainly to resource competition (for light and water, for example), we would expect a similar or stronger effect on the plants under *Capparis*, but this was not the case. Hence, it is possible that *Leucaena* trees release allelopathic substances that do not affect germination but do affect the development of the seedlings, reducing their ability to acquire resources and, thus, their survival and growth. There are several difficulties inherent to separating the effects of resource competition from those of allelopathy [42], but the effects of allelopathy, and not water or light limitation, are likely important in this case. Regardless, determining the relative role of each mechanism requires experiments designed with this particular objective (see [43]).

The balance of the facilitative and competitive aspects of the interaction between *Capparis* and *Erythrina* seedlings may explain the neutral outcome of the effects of *Capparis* on *Erythrina*. In dry ecosystems, plants are exposed to high temperatures and desiccating conditions, and the establishment of seedlings has often been reported to be mostly restricted to shady sites, under the canopy of the so-called “nurse plants” [44]. However, these nurse plants can simultaneously negatively affect seeding growth by reducing the availability of light and soil water [45]. It is possible that *Capparis* provides shade and acts as a nurse plant that facilitates the initial establishment of *Erythrina* during a phase when the seedlings are more fragile. However, the development of the seedlings may be increasingly inhibited by the amount of shade as the seedlings grow because plants that grow under drier conditions are predicted to be less shade tolerant [46].

The negative effect of *Leucaena* on *Erythrina* is aggravated by the presence of *Capparis*, which is the native plant that is most often observed growing under *Leucaena*. When associated with *Leucaena*, *Capparis* decreases the probability of *Erythrina* survival and the lowest survival of *Erythrina* was observed in the treatment that combined both the exotic and native species. The pattern of seedling growth was the same as the pattern observed for survival. The few seedlings that survived under *Leucaena* plus *Capparis* were shorter than those under *Leucaena* and much shorter than those in the control or growing under *Capparis* alone.

If this effect mentioned in the paragraph above was due solely to crowding, the effect of the combination of exotic and native presence would be the addition of the isolated effects that we have observed independently. However, what we have observed was that the negative effect on *Erythrina* was stronger when *Leucaena* and *Capparis* were combined when compared to the independent effects added together. Besides that, given that *Capparis* is evergreen and its crown is denser, shading the environment more (see Fig 1), we would expect the effect of *Capparis* to be more negative than the effect of *Leucaena*. Therefore crowding effect cannot explain the interaction effect of the presences of *Capparis* and *Leucaena* on *Erythrina*.

The results indicate that the presence of *Capparis* under the *Leucaena* canopy further inhibits the establishment of *Erythrina*. It is possible that *Leucaena* indirectly affects *Erythrina* via *Capparis* as a mediator species, debilitating the seedlings to such an extent that they cannot cope with a new competitor. Whenever there are many species interacting, the negative effects of one species on another can decrease the latter’s negative effect on a third species, a phenomenon known as indirect facilitation [3]. While other studies have found this positive indirect interactions, this is not the mechanism behind *Leucaena* invasion. The negative effect of *Leucaena* on *Erythrina* might change the effect of *Capparis* from positive or neutral to negative; therefore, the negative effects of *Leucaena* are both direct and indirect mediated by other native species. Complex interactions like indirect effects are often neglected in studies of biological invasion [7]. To our knowledge, this is the first study in which it was experimentally demonstrated that the balance of the interactions between native species is altered in the presence of an exotic invader, further promoting the exclusion of other native species via indirect effects.

Interactions among plants influence the recovery of a community from disturbance events [47], and indirect effects must be considered when attempting to predict how native plants respond to invasion [10]. Many of the areas in Fernando de Noronha that are now dominated by *Leucaena* and *Capparis* were deforested decades ago. The presence of pioneer species has been suggested to facilitate the establishment of seedlings of other species, especially in stressful environments such as dry ecosystems, as observed in the caatinga [48]. However, when the pioneer is an exotic species that inhibits the establishment of seedlings, instead of facilitating establishment, succession may be arrested for several decades [49], even if seed arrival is sufficient [38]. From a restoration and conservation viewpoint, this situation is alarming because associations of *Leucaena* and *Capparis* are among the most common associations found on Fernando de Noronha Island. Our findings highlight the importance of indirect effects of biological interactions during the invasion process and indicate that this information should be incorporated to yield more effective management strategies.

## Supporting Information

S1 Table List of models compared for field experiment of *Erythrina velutina* germination. The models were binomial generalised linear mixed models (GLMMs) with germination as the response variable, the presence of *Leucaena leucocephala* (leu), *Capparis flexuosa* (cap) or litter (lit) and their interactions as fixed predictor variables and the blocks as a random variable. ■ indicate that factor or interaction is considered in the model. Selected models (*ΔAIC* < 2) are highlighted in gray. *ΔAIC* = AIC for each model—AIC for the best model (Model 05).
(PDF)

S1 Dataset Raw data from *Leucaena leucocephala* abundance rank in Fernando de Noronha island. (CSV)

S2 Dataset Raw data from native species ocurrence in invaded and non invaded plots in Fernando de Noronha island. (CSV)

S3 Dataset Raw data from germination nursery experiment of *Erythrina velutina*: soil. (CSV)

S4 Dataset Raw data from germination nursery experiment of *Erythrina velutina*: litter. (CSV)

S5 Dataset Raw data from germination field experiment of *Erythrina velutina*. (CSV)

S6 Dataset Raw data from field experiment of *Erythrina velutina* seedlings survival. (CSV)

S7 Dataset Raw data from field experiment of *Erythrina velutina* seedlings growth. (CSV)

S1 Analysis Code Native species occurence analysis: R code. (R)

S2 Analysis Code Nursery germination analysis: R code. (R)

S3 Analysis Code Field germination analysis: R code. (R)

S4 Analysis Code Field survival analysis: R code. (R)

S5 Analysis Code Field growth analysis: R code. (R)
