# Supplementary material for: Making a Bad Situation Worse: An Invasive Species Altering the Balance of Interactions between Local Species
Source: PLoS One. 2016 Mar 24;11(3):e0152070. doi: 10.1371/journal.pone.0152070 (PMC4807039; doi:10.1371/journal.pone.0152070)
Supplement: S1 Table — The models were binomial generalised linear mixed models (GLMMs) with germination as the response variable, the presence of Leucaena leucocephala (leu), Capparis flexuosa (cap) or litter (lit) and their interactions as fixed predictor variables and the blocks as a random variable. ■ indicate that factor or interaction is considered in the model. Selected models (ΔAIC < 2) are highlighted in gray. ΔAIC = AIC for each model—AIC for the best model (Model 05). (PDF) [file pone.0152070.s001.pdf]

S1 Table

List of models compared for field experiment of *Erythrina velutina* germination. The models were binomial generalised linear mixed models (GLMMs) with germination as the response variable, the presence of *Leucaena leucocephala* (leu), *Capparis flexuosa* (cap) or litter (lit) and their interactions as fixed predictor variables and the blocks as a random variable. ■ indicate that factor or interaction is considered in the model. Selected models ($\Delta AIC < 2$) are highlighted in gray. ΔAIC = AIC for each model - AIC for the best model (Model 05).

**Model**	**Parameters included**							ΔAIC
	**leu**	**cap**	**lit**	**leu:cap**	**cap:lit**	**leu:lit**	**leu:cap:lit**	
full	■	■	■	■	■	■	■	4.4
01	■	■	■	■	■	■	□	3.0
02	■	■	■	■	■	□	□	1.1
03	■	■	■	■	□	■	□	1.9
04	■	■	■	□	■	■	□	4.6
05	■	■	■	■	□	□	□	0.0
06	■	■	■	□	□	■	□	3.6
07	■	■	■	□	■	□	□	2.8
08	■	■	■	□	□	□	□	1.7
09	■	■	□	□	□	□	□	3.5
10	■	□	■	□	□	□	□	0.3
11	□	■	■	□	□	□	□	11.6
12	□	□	■	□	□	□	□	10.2
13	□	■	□	□	□	□	□	13.4
14	■	□	□	□	□	□	□	2.1
null	□	□	□	□	□	□	□	12.0
